# Metastatic pulmonary calcification: high-resolution computed tomography findings in 23 cases

**DOI:** 10.1590/0100-3984.2016-0123

**Published:** 2017

**Authors:** Luciana Camara Belém, Carolina A. Souza, Arthur Soares Souza Jr., Dante Luiz Escuissato, Bruno Hochhegger, Luiz Felipe Nobre, Rosana Souza Rodrigues, Antônio Carlos Portugal Gomes, Claudio S. Silva, Marcos Duarte Guimarães, Gláucia Zanetti, Edson Marchiori

**Affiliations:** 1 MD, Universidade Federal do Rio de Janeiro (UFRJ), Rio de Janeiro, RJ, Brazil.; 2 MD, PhD, Ottawa Hospital Research Institute, University of Ottawa, Canada.; 3 MD, PhD, Faculdade de Medicina de São José do Rio Preto (Famerp) and Ultra X, São José do Rio Preto, SP, Brazil.; 4 MD, PhD, Universidade Federal do Paraná (UFPR), Curitiba, PR, Brazil.; 5 MD, PhD, Universidade Federal de Ciências da Saúde de Porto Alegre (UFCSPA), Porto Alegre, RS, Brazil.; 6 MD, PhD, Hospital Universitário, Universidade Federal de Santa Catarina (UFSC), Florianópolis, SC, Brazil.; 7 MD, PhD, Universidade Federal do Rio de Janeiro (UFRJ) and Instituto D'Or de Pesquisa e Ensino, Rio de Janeiro, RJ, Brazil.; 8 MD, Hospital Beneficência Portuguesa and Med Imagem, São Paulo, SP, Brazil.; 9 MD, MSc, Facultad de Medicina Clinica Alemana, Universidad del Desarrollo, Santiago, Chile.; 10 MD, PhD, A.C.Camargo Cancer Center, São Paulo, SP, and Universidade Federal do Vale do São Francisco (Univasf), Petrolina, PE, Brazil.; 11 MD, PhD, Universidade Federal do Rio de Janeiro (UFRJ), Rio de Janeiro, RJ, Brazil.

**Keywords:** Metastatic pulmonary calcification, Computed tomography, Metabolic diseases, Pulmonary diseases

## Abstract

**Objective:**

The aim of this study was to evaluate the high-resolution computed tomography (HRCT) findings in patients diagnosed with metastatic pulmonary calcification (MPC).

**Materials and Methods:**

We retrospectively reviewed the HRCT findings from 23 cases of MPC [14 men, 9 women; mean age, 54.3 (range, 26-89) years]. The patients were examined between 2000 and 2014 in nine tertiary hospitals in Brazil, Chile, and Canada. Diagnoses were established by histopathologic study in 18 patients and clinical-radiological correlation in 5 patients. Two chest radiologists analyzed the images and reached decisions by consensus.

**Results:**

The predominant HRCT findings were centrilobular ground-glass nodules (*n* = 14; 60.9%), consolidation with high attenuation (*n* = 10; 43.5%), small dense nodules (*n* = 9; 39.1%), peripheral reticular opacities associated with small calcified nodules (*n* = 5; 21.7%), and ground-glass opacities without centrilobular ground-glass nodular opacity (*n* = 5; 21.7%). Vascular calcification within the chest wall was found in four cases and pleural effusion was observed in five cases. The abnormalities were bilateral in 21 cases.

**Conclusion:**

MPC manifested with three main patterns on HRCT, most commonly centrilobular ground-glass nodules, often containing calcifications, followed by dense consolidation and small solid nodules, most of which were calcified. We also described another pattern of peripheral reticular opacities associated with small calcified nodules. These findings should suggest the diagnosis of MPC in the setting of hypercalcemia.

## Abstract (pt)

**Objetivo:**

O objetivo deste estudo foi avaliar os achados de tomografia computadorizada de alta resolução (TCAR) em pacientes com diagnóstico de calcificação pulmonar metastática (CPM).

**Materiais e Métodos:**

Revisamos, retrospectivamente, os achados na TCAR de 23 casos de CPM [14 homens e 9 mulheres; idade média, 54,3 (intervalo, 26-89) anos]. Os pacientes foram examinados entre 2000 e 2014 em nove hospitais terciários no Brasil, Chile e Canadá. O diagnóstico foi estabelecido por estudo histopatológico em 18 pacientes e correlação clinicorradiológica em 5 pacientes. Dois radiologistas de tórax analisaram as imagens e chegaram a decisões por consenso.

**Resultados:**

Os achados predominantes na TCAR foram nódulos centrolobulares em vidro fosco (*n* = 14; 60,9%), consolidação com alta atenuação (*n* = 10; 43,5%), pequenos nódulos densos (*n* = 9; 39,1%), opacidades reticulares periféricas associadas com pequenos nódulos calcificados (*n* = 5; 21,7%) e opacidades em vidro fosco sem nódulos centrolobulares em vidro fosco (*n* = 5; 21,7%). Calcificação vascular na parede torácica foi encontrada em quatro casos e derrame pleural foi observado em cinco casos. As anormalidades foram bilaterais em 21 casos.

**Conclusão:**

CPM manifestou-se com três padrões principais na TCAR. O aspecto mais comum foram os nódulos centrolobulares em vidro fosco, frequentemente contendo calcificações, seguido de consolidação densa e de pequenos nódulos sólidos, a maioria deles calcificada. Também descrevemos o padrão de opacidades reticulares periféricas associadas a pequenos nódulos calcificados. Esses achados devem sugerir o diagnóstico de CPM no contexto de hipercalcemia.

## INTRODUCTION

Metastatic pulmonary calciﬁcation (MPC) is a metabolic lung disease characterized by the deposition of calcium in normal lung tissue under conditions that directly or indirectly result in hypercalcemia^(1-3)^. MPC is known to be a long-term complication of chronic renal failure with secondary hyperparathyroidism. Other causes include primary hyperparathyroidism, excessive exogenous administration of calcium and vitamin D, massive osteolysis from metastases or multiple myeloma, orthotopic liver transplantation, and heart surgery^(1,4-6)^.

The disease process is characterized by interstitial deposition of calcium salts, predominantly in the alveolar epithelial basement membranes^(7,8)^. Although histological changes of MPC are encountered on autopsy in 60-75% of patients who received hemodialysis^(1,7,9,10)^, the condition is much less commonly diagnosed antemortem. This situation is probably the result of the poor sensitivity of standard chest radiographs for the identification of small calcifications, the lack of awareness among clinicians of the imaging manifestations of MPC, and the benign clinical course of the disease^(11,12)^.

The clinical manifestations of MPC are usually minimal; when present, symptoms are nonspecific^(13)^ and include dyspnea and non-productive cough. Acute respiratory failure has been described in patients with MPC, although it is rare^(14-17)^. Thus, diagnosis is usually first suspected and relies mainly on imaging findings. The relative stability of pulmonary infiltrates and their persistence despite treatment, in the presence of hypercalcemia, are important diagnostic clues^(12,18)^. Plain radiographs, however, are often normal or demonstrate non-specific findings^(9)^. Computed tomography (CT), particularly high-resolution computed tomography (HRCT), is much more accurate than chest radiography^(9)^ for the detection and characterization of parenchymal opacities and calcification, and can depict even subtle abnormalities. HRCT may demonstrate characteristic findings that allow a presumptive diagnosis of MPC, thereby obviating the need for lung biopsy^(19-21)^. The aim of this study was to describe the spectrum of HRCT findings of MPC in a heterogeneous group of patients.

## MATERIALS AND METHODS

Our institutional review board approved this study and waived the requirement for informed patient consent. All data used in this study were anonymized. This retrospective study included 23 patients diagnosed with MPC. The patients were examined between 2000 and 2014 in nine tertiary hospitals in Brazil, Chile, and Canada. The diagno sis of MPC was based on medical history, clinical course, and imaging findings. Lung biopsy was performed in 17 cases and case autopsy results were available in one case. Information regarding patients' demographic characteristics, underlying disease, and laboratory test findings was obtained by retrospective review of patients' charts.

Chest CT examinations were performed using a variety of helical scanners, as different hospitals were involved in this study. In initial studies, HRCT images were obtained at full inspiration with 1-2-mm slice thickness at 5-10-mm intervals and reconstructed using a high-spatial-frequency algorithm. The most recent CT examinations were performed using helical acquisition and reconstructed with 1-2-mm slice thickness and 1-2-mm intervals using a high-spatial-frequency algorithm. The acquisition time was 0.5-1 s per rotation, peak voltage was 120 kVp, modulated tube current was 100-400 mA, pitch was 1, and matrix was 512 × 512 pixels. The images were reviewed using mediastinal (width, 350-450 HU; level, 10-20 HU) and lung (width, 1,200-1,600 HU; level, -500 to -700 HU) window settings. Two chest radiologists with more than 15 years of experience independently reviewed the images, and final assessment was achieved by consensus.

The CT images were evaluated to determine the presence and distribution of nodules, consolidation, ground-glass opacities, reticular opacities, and associated findings, such as pleural effusion or chest wall vascular calcification. Consolidation was defined as an opacity that obscured vessel margins and airway walls, with or without air bronchograms. Consolidations were designated as dense when they presented density greater than that of soft tissues in the mediastinal window or scattered foci of calcification. Ground-glass opacities appear as hazy increased opacity of lung, with preservation of bronchial and vascular margins. A nodule was defined as a rounded or irregular opacity that was well or poorly defined and ≤ 3 cm in diameter. Nodules were classified as small (diameter < 10 mm) or large (diameter > 10 mm) and categorized according to their attenuation as calcified, ground-glass (not obscuring underlying bronchial and vascular margins), or solid (presenting homogenous soft-tissue attenuation). Reticular opacities are a collection of innumerable small linear opacities with an appearance resembling a net. The definition of HRCT findings followed the Glossary of Terms for Thoracic Imaging proposed by the Fleischner Society^(22)^.

The distribution of abnormalities was categorized as bilateral or unilateral and as predominantly in the upper or lower lung lobes, or as diffuse. The tracheal carina was used as the division between the upper and lower zones of the lungs when determining the predominant distribution^(20)^.

### Statistical analysis

All data were extracted from two forms containing demographic information and imaging features and entered into Microsoft Excel 2008 software. Descriptive analyzes were used to describe the features of the data and were presented as mean ± standard deviation and frequency (expressed as percentages).

## RESULTS

The sample included 23 patients (14 men, 9 women) with a mean age of 54.3 (range, 26-89) years. Most (*n* = 21; 91.3%) of the patients had chronic renal disease; one patient had MPC secondary to multiple myeloma and one had normal calcium levels and no evidence of an underlying metabolic or renal abnormality, with a biopsy-proven diagnosis of idiopathic MPC.

**Figure 1:**
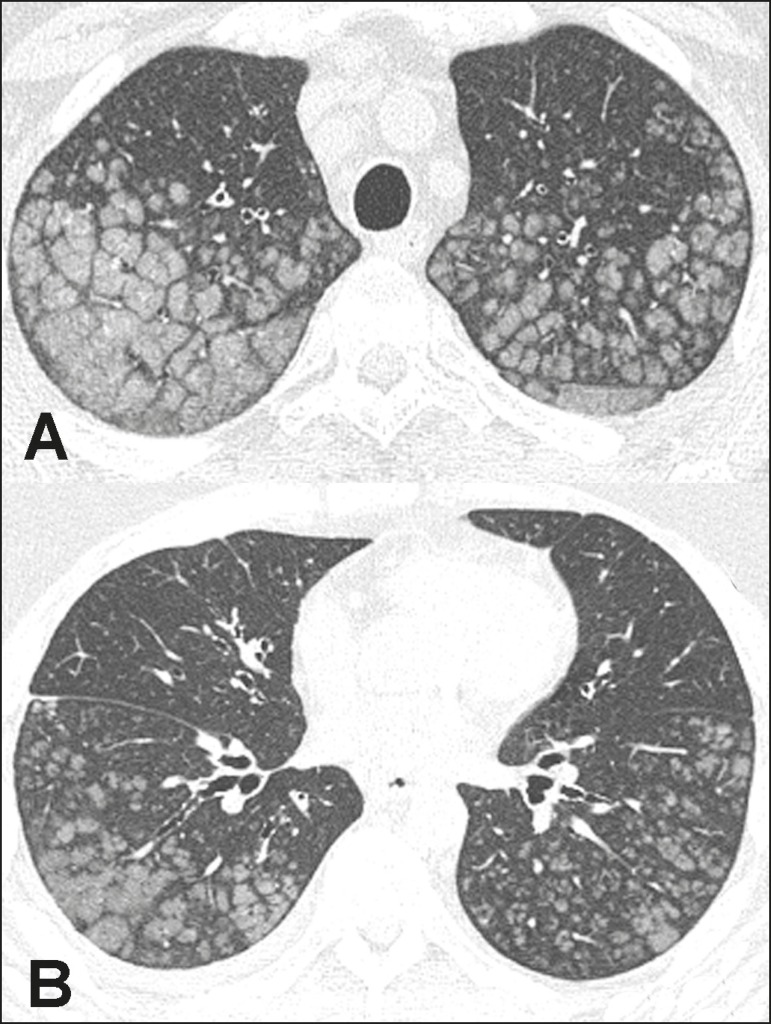
37-year-old woman with biopsy-proven metastatic pulmonary calcification due to end-stage renal disease. **A:** HRCT at the level of the upper lobes shows bilateral ill-defined centrilobular ground-glass nodular opacities, with confluence. **B:** Image obtained at the level of the lower lobes shows similar, but less extensive, findings.

**Figure 2:**
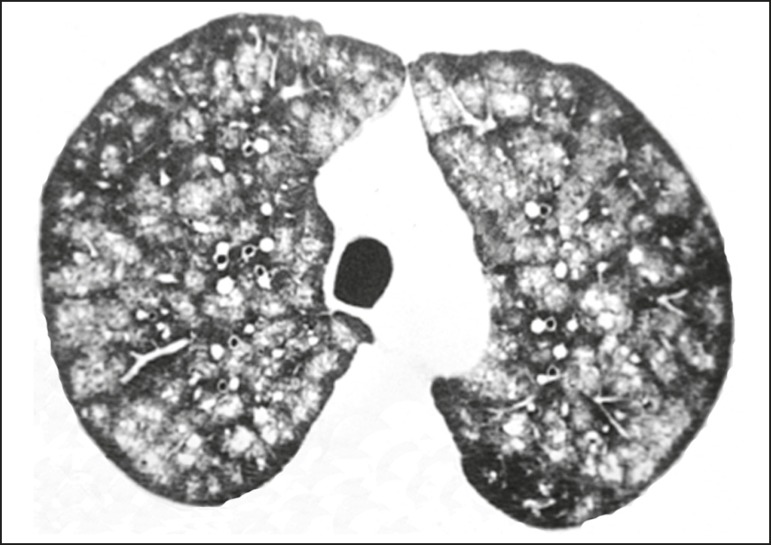
67-year-old man with biopsy-proven idiopathic metastatic pulmonary calcification. HRCT at the level of the upper lobes shows bilateral ill-defined centri-lobular ground-glass nodules. Mediastinal window (not shown) demonstrated punctiform calcification in some nodules and diffuse calcification in others.

HRCT findings are summarized in Table 1. The main HRCT pattern, found in 14 (60.9%) patients, was centrilobular ground-glass nodular opacities (Figure 1). Punctate calcification within nodular opacities was observed in 6 (42.6%) of these 14 patients (Figure 2). In the remaining eight (57.1%) cases, the ground-glass nodules showed no calcification. In nine (31.9%) patients, centrilobular ground-glass nodules were the only imaging finding.

**Table 1:** HRCT findings in patients with metastatic pulmonary calcification.

Finding	N	%
Airspace ground-glass nodular opacities	14	60.9%
Dense consolidation	10	43.5%
Small dense nodules	9	39.1%
Ground-glass opacities without centrilobular nodules	5	21.7%
Reticular opacities	5	21.7%
Pleural effusion	5	21.7%
Chest wall vascular calcification	4	17.4%

**Figure 3:**
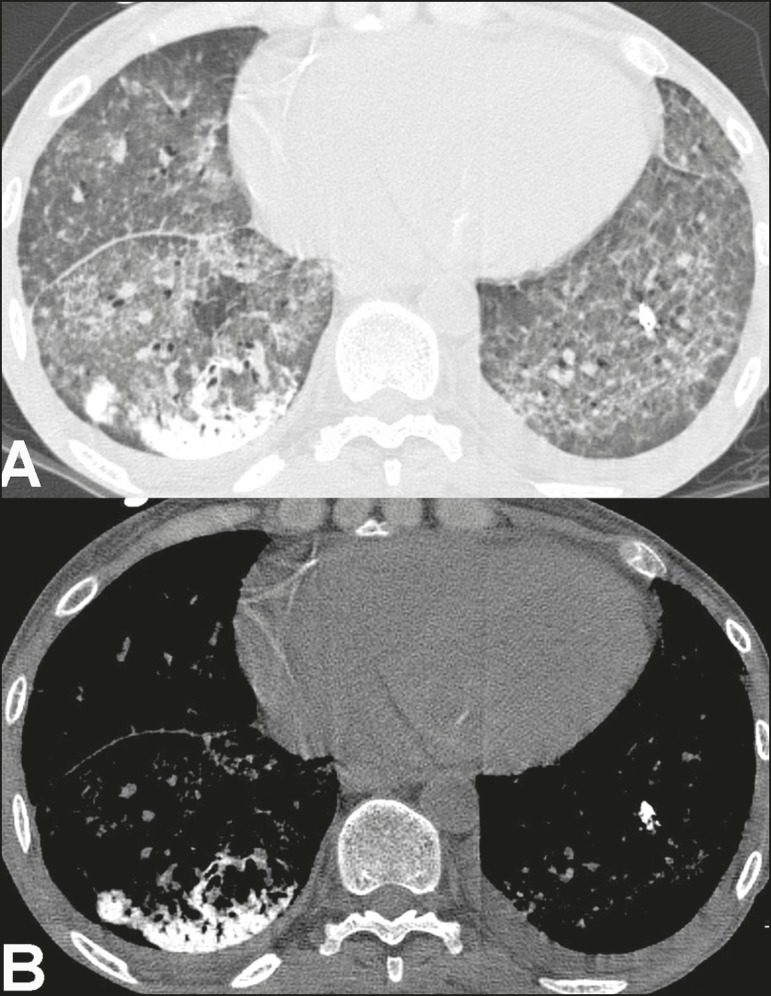
40-year-old woman with biopsy-proven metastatic pulmonary calcification. **A:** HRCT at the level of the lower lobes shows diffuse bilateral ground-glass opacities and dense consolidation in the posterior right lower lobe. **B:** Image obtained at the same level with mediastinal window settings demonstrates calcification within the area of consolidation, as well as scattered punctate foci of calcification. Note also small bilateral pleural effusion.

Consolidation with high attenuation (dense consolidation) was observed in 10 (43.5%) cases (Figure 3) and was typically diffusely dense, apart from one case that showed punctuate foci of calcification. Consolidation was associated with other patterns in most (*n* = 8) cases, but it was the only finding in two patients.

**Figure 4:**
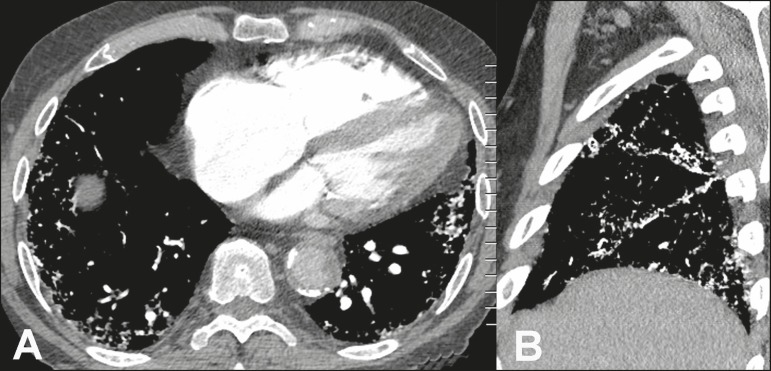
89-year-old man with biopsy-proven metastatic pulmonary calcification due to end-stage renal disease. **A:** Axial image obtained with mediastinal window settings at the level of the lower lobes shows multiple small, dense, subpleural nodules. **B:** Sagittal image obtained with mediastinal window settings demonstrates the peripheral distribution of the nodules, in a subpleural location and adjacent to the fissures.

**Figure 5:**
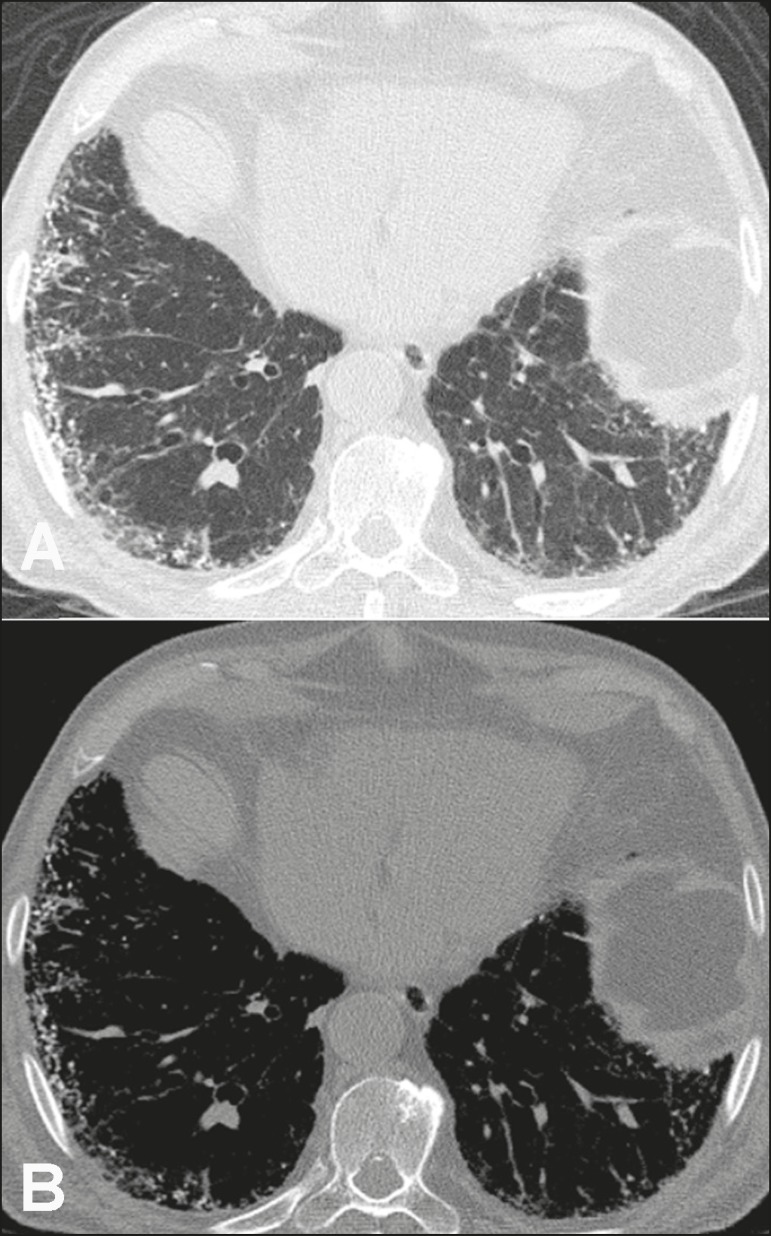
85-year-old man with biopsy-proven metastatic pulmonary calcification due to end-stage renal disease. **A:** HRCT at the level of the lower lobes shows multiple small, dense, peripheral nodules associated with reticular opacities. **B:** Image obtained at the same level with mediastinal window settings shows scattered foci of calcification along the nodules.

**Figure 6:**
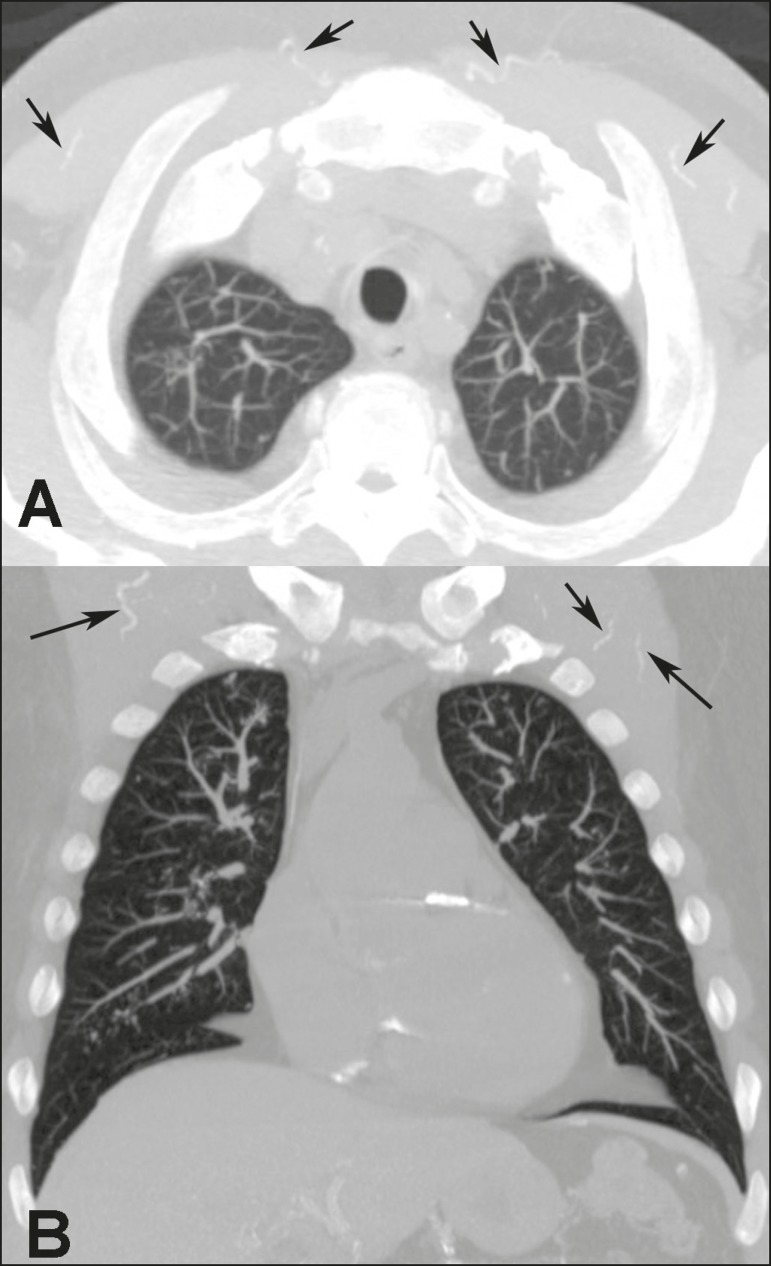
55-year-old man with biopsy-proven metastatic pulmonary calcification due to end-stage renal disease. **A:** Axial maximum intensity projection (MIP) image demonstrates calcification along the vessels of the chest wall (arrows). **B:** Coronal MIP image shows calcification along the coronary and abdominal arteries, and in the vessels of the chest wall (arrows).

Small nodules, most of which were calcified, were observed in nine (39.1%) patients (Figure 4). These nodules were the only finding in one case. Peripheral reticular opacities associated with small calcified nodules were observed in five (21.7%) patients (Figure 5). Ground-glass opacities without centrilobular ground-glass nodular opacity were also found in five (21.7%) patients. Calcification in the vessels of the chest wall was seen in four (17.4%) cases (Figure 6) and pleural effusion was observed in five (21.7%) patients.

Abnormalities were bilateral in 21 (91.3%) patients, with right lung predominance in 4 of these cases. In the two cases with unilateral disease, the right lung was involved. The disease was diffuse in five (21.7%) patients and predominated in the lower and upper lobes in nine (39.1%) patients each.

## DISCUSSION

CT of the chest has been the subject of a series of recent publications in the Brazilian radiological literature^(23-31)^. In our study, the main HRCT finding of MPC was centrilobular ground-glass nodules, observed in 60.9% of patients. Calcification within these nodules was found in 42.6% of cases. Dense consolidation was the second most common pattern (43.5%), followed by small nodules, most of them calcified (39.1%). Peripheral reticular opacities associated with small calcified nodules were observed in 21.7% of patients. To our knowledge, this pattern was not previously described in the literature. Pleural effusion was found in five (21.7%) of our patients. Few studies have reported the HRCT manifestations of MPC. Nonetheless, three CT patterns have been described and concur with our findings: multiple diffuse calcified nodules, diffuse or patchy areas of ground-glass opacity or consolidation, and confluent dense parenchymal consolidation with lobar distribution^(19-21)^. Calcification is common and may be punctate within nodular opacities, ringlike, or diffuse, involving the entire nodule or area of consolidation^(20,21,32-35)^. Of these, the pattern of centrilobular ground-glass nodules with or without foci of calcification^(32)^ has been described as most common, as corroborated by our results. In a series of seven patients^(20)^, the most common CT finding was multiple fluffy, poorly defined nodules, present in all patients and calcified in 57% of cases, followed by diffuse ground-glass opacities in three (43%) patients and consolidation in two (28%) patients.

In our study, abnormalities were bilateral in most patients. Interestingly, right-side predominance was observed in four of these cases, and only the right side was affected in two cases of unilateral disease. Regarding zonal distribution, abnormalities were equally predominant in the lower and upper lung zones (39% each) and diffuse in 21% of cases. These results contrast with the series reported by Hartman et al.^(20)^ in which nodules were more commonly diffuse or showed upper lung predominance (43% each); a predominantly lower-lung zonal distribution was found in only one (14%) patient.

Vascular calcification in the chest wall has been reported as a common ancillary finding in patients with MPC, and the combination of pulmonary and vascular calcification is considered to have diagnostic value, narrowing the differential diagnosis of causes of pulmonary calcification^(20,21,34,36)^. Hartman et al.^(20)^ found this manifestation in 86% of cases, and observed calcification within the left atrial wall in one case. In contrast, chest wall vascular calcification was found in a minority of our cases.

Our study has several limitations. First, it was retrospective in nature and not all cases were confirmed histologically, which inherently limited clinical-radiological-pathological correlation. Second, HRCT techniques varied over the 14-year study period and among the institutions involved. We do not believe, however, that this variation altered our observations or results. Thus, despite these limitations, our results demonstrate the HRCT manifestations of MPC in the largest series of patients reported to date.

In conclusion, MPC manifested with three main patterns on HRCT, most commonly centrilobular ground-glass nodules, often containing calcifications, followed by dense consolidation and small solid nodules, most of which were calcified. We also described another pattern of peripheral reticular opacities associated with small calcified nodules. These findings should indicate the diagnosis of MPC in the setting of hypercalcemia. Awareness of the spectrum of MPC manifestations is crucial for radiologists, as the diagnosis is often first suggested and relies on HRCT findings.
